# The potential of hemp roots, microgreens and leaves for human nutrition

**DOI:** 10.1186/s42238-025-00319-2

**Published:** 2025-10-07

**Authors:** Harpal Singh, Tobias Kretzschmar, Ashley Dowell, Nadia Toppler, Andrew Kavasilas, Jos Mieog

**Affiliations:** 1 Southern Cross University, Lismore, Australia; 2 Kavasil Pty Ltd, Nimbin, Australia

**Keywords:** *Cannabis*, Cannabinoids, Nutrients, Microgreens, Food

## Abstract

*Cannabis sativa* L. has been utilised for food, fibre and medicine for millennia. However, historical legal constraints due to the presence of psychoactive cannabinoids have limited the use of the plant for human nutrition. For instance, in Australia, only dehulled hemp seeds and their derivatives have been available for human consumption since 2017, while the nutritional potential of other tissues remains overlooked. In this study, hemp leaves, roots and microgreens from cultivar HAN NE were analysed to determine their nutritional properties, including minerals, vitamins, terpenes, amino acids, and proximate data. Furthermore, cannabinoid contents were determined in target tissues, together with the expression of key genes involved in cannabinoid biosynthesis. Results highlighted the potential role of hemp leaves and microgreens in human nutrition, being able to provide a good source of protein, vitamin E, and various other essential micronutrients and minerals. The cannabinoid pathway was found to be active in microgreens and young leaves but not in roots, reflected by the measured cannabinoid concentrations in these tissues, which emphasised the importance of picking suitable cultivars for leaf and microgreens. Additionally, arsenic accumulation was observed in the roots, highlighting an important aspect of using roots for human nutrition.

**Supplementary Information:**

The online version contains supplementary material available at 10.1186/s42238-025-00319-2.

## Introduction

*Cannabis sativa* L., a herbaceous annual plant species in the *Cannabaceae* family, is native to central Asia and the Indian subcontinent (Filipiuc et al. 2021) but, being adaptive to both tropical and temperate environmental conditions, has spread globally through human intention (Karche and Singh 2019; Clarke and Merlin 2016). The plant with its distinctive palmate compound leaves is perhaps best known for its psychoactive effects due to the accumulation of the cannabinoid Δ9-tetrahydrocannabinol (THC). THC accumulates in the trichomes that are most abundant in female inflorescence, which has long been used for recreational and medicinal purposes (Jalali et al. 2019). The terpenoids, which also accumulate in the trichomes, give *C. sativa* its distinctive smell (Mahlberg and Kim 2004; Russo 2011).

Humans have a long history with *C. sativa*, having cultivated the plant since prehistoric times for a variety of purposes including fibre, food, medicine and rituals (Clarke and Merlin 2016). As a multi-use and phychoactive plant, *C. sativa* has been given common names reflecting not only the purpose it is cultivated for but also based on how it is regulated (Chouvy 2025). Plants grown for psychoactive uses are known as medicinal cannabis or marijuana and generally have high THC content (up to 30% of dried flower bud) (Small and Marcus 2002). Plants commercially grown for non-cannabinoid purposes (seed, fibre) are known as (low-THC or industrial) hemp and are nowadays strictly regulated to ensure (very) low levels of THC (< 0.2–1% by dry weight) (Small 2015; Mead 2019). Hemp seed was one of the major grain crops grown in China, next to millet, rice, soybean, and barley (Li 1974). In Europe, the plant was introduced around 2000 years ago and has been widely used for clothing, paper, ropes, fish nets, and canvas sails (Li 1974; Ranalli and Venturi 2004; Addlesperger 2015). However, literature regarding the use of hemp as food in Europe is scarce.

In the twentieth century, the psychoactive nature of *C. sativa* and its use for recreational purposes were deemed increasingly problematic as the value of the illicit international drug industry surpassed the value of hemp markets (Small and Marcus 2002), and led to calls for the prohibition of *C. sativa*. As a consequence, many countries started to ban or strictly regulate the plant, and the UN placed it on Schedule IV around the mid-twentieth century (Crocq 2020), as the drug-related risks were considered to outweigh its medicinal, nutritional and industrial benefits (Lohman and Barrett 2020). In many countries, including the USA and Australia, no distinction was made between hemp and marijuana and all *C. sativa* cultivation became prohibited. Other countries, including Germany, Great Britain, and Canada, understood the potential of hemp and and allowed cultivation to continue under strict regulation (Thedinger 2005). Over time, many countries (incl. the USA and EU) again legalised and regulated the production of hemp (Brady 2003) as its potential value became more widely re-recognised. For instance, in the USA, the Agriculture Improvement Act of 2018 excluded hemp from the definition of marijuana, allowing its cultivation (Mead 2019). In Europe, the cultivation of hemp has increased significantly over the last decade, with France being the leading cultivator, followed by Germany and the Netherlands (Hemp production in the EU. Available online 2024).

Cultivation of hemp for fibre and seeds in Australia has been possible again since 2008 (Lewis and Flood 2021). However, no sales for human nutrition were allowed until the Food Standard Australia New Zealand (FSANZ) approved the use of dehulled seeds from hemp varieties for human consumption in (2017), setting strict limits for detectable cannabinoids, including CBD, THC and their acidic precursors (Hemp and Seeds as Food. Available online 2024). Apart from seeds, however, no other hemp tissues are currently approved for human consumption in Australia, despite the potential beneficial role they could play in this space.

Green leafy vegetables are considered healthy, are recognised as good sources of essential nutrients and have been shown to reduce the risk of chronic diseases (Hung et al. 2004; Nikolić et al. 2008). Microgreens have been shown to have various beneficial functional nutrients such as antioxidants, vitamins, minerals and phenolic compounds with higher concentrations when compared to mature leaves and seeds (Janovská et al. 2010). In addition to their nutritional values, microgreens are recognised as health-promoting and disease-preventing functional foods (Mir et al. 2017). Hemp seeds contain a high content of polyunsaturated fatty acids (PUFAs), especially ω−3 and ω−6 fatty acids (Banskota et al. 2022) and the health benefits of PUFAs have been reviewed (Kapoor et al. 2021). Hemp sprouts have been shown to have various health-promoting bioactive compounds such as polyphenols, including flavonoids and flavonols (Frassinetti et al. 2018). Thus, it is highly likely that hemp leaves and microgreens can be utilised as good sources of PUFAs, amino acids, and polyphenols for human nutrition. Hemp leaves could also be dried and used to make herbal tea. Hemp roots are not appealing as a direct source of food, but may be used as a coffee substitute once roasted or in the preparation of herbal teas. Various health benefits attributed to the chemical composition of hemp roots are documented, including anti-inflammatory (Huang et al. 2023; Ferrini et al. 2022), antidiabetic (Kim et al. 2023), antioxidant (Ferrini et al. 2022), analgesic, and antigout (Ryz et al. 2017). Notwithstanding the potential of various hemp tissues as food, the seeds currently represent the only consumed part of the plant in Australia, in the form of dehulled seeds, flour, butter, cold-pressed oil, and hemp seed protein.

This study focused on acquiring compositional information on hemp roots (HR), hemp microgreens (HMG) and young hemp leaves (YHL) to assess their potential role in human nutrition. The assessments focused on nutrients, anti-nutrients, heavy metals, natural toxicants, cannabinoids, terpenoids, phenolic compounds, microbiological contaminants, amino acids, and proximate analysis. This study further determined the expression of several genes involved in the cannabinoid biosynthesis pathway in these vegetative tissues to improve the understanding of the potential for cannabinoid accumulation. Results are discussed in terms of potential benefits and barriers to the use of hemp vegetative tissues for the purpose of human nutrition, with the hope that it can contribute to the process of making hemp vegetative tissues available for human consumption in Australia and the rest of the world.

## Materials and methods

### Plant material

Seeds of HAN NE, a low THC hemp of Chinese origin, were obtained from the Southern Cross University *Cannabis* Germplasm Collection (accession ID AK2300002_02_SH) and were cultivated under NSW Industrial Hemp Licence 52204 to produce the plant material for the various analyses.

### Production of hemp roots (HR), young hemp leaves (YHL), hemp microgreens (HMG) and inflorescence

**Fig. 1:**
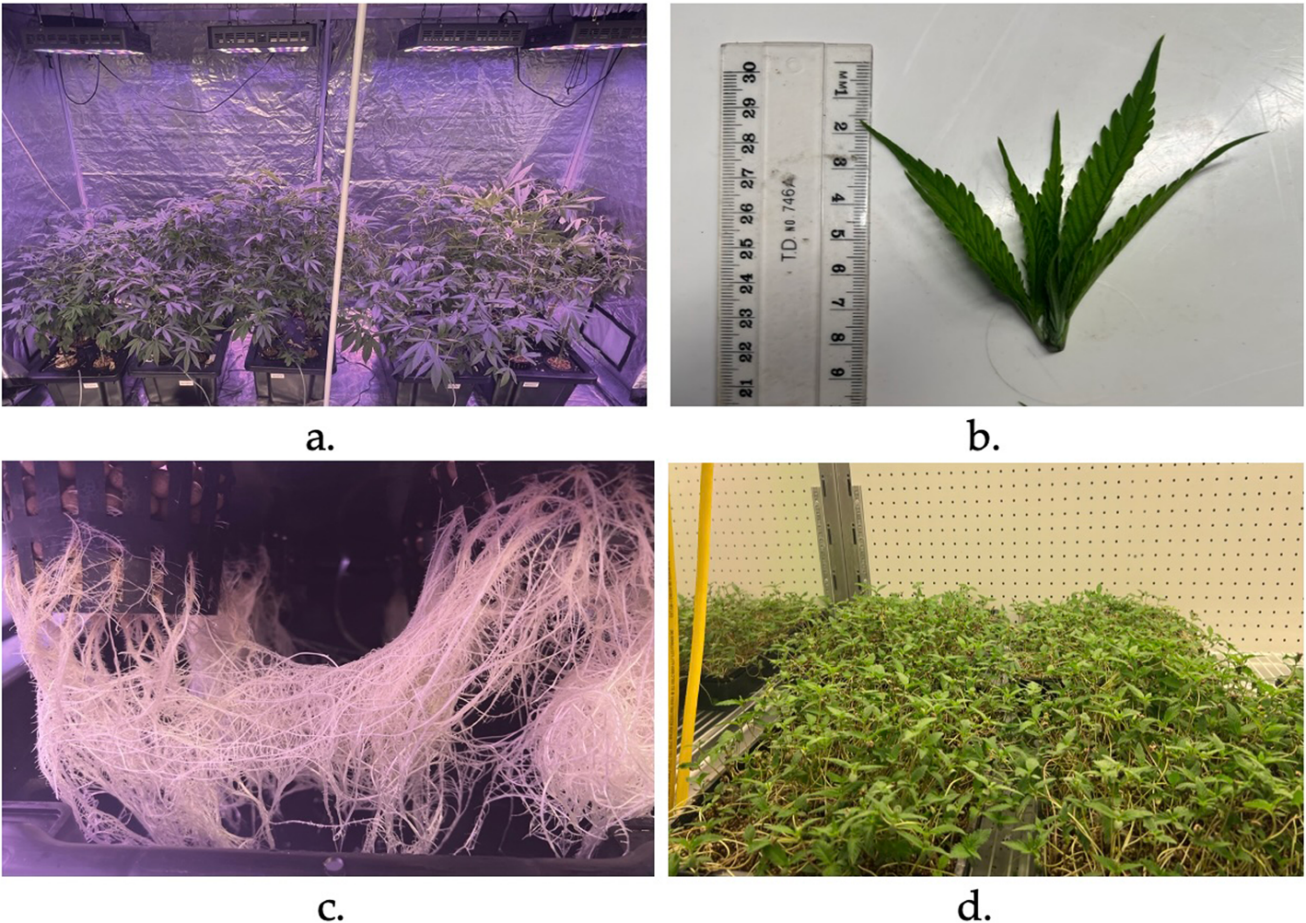
Figure showing the sample production setup. (**a**) five hydroponic setups in a tent to produce YHL and HR for analysis grown under vegetative cycle (24 h light), (**b**) an example of leaf tip samples taken for analyses, (**c**) growth of the HR in hydroponic tubs at the time of harvest, all root outside the net pot was harvested (**d**) HMG at the time of harvesting after growth of 10 days (four days in the dark and six days under light)

The seeds were germinated in HIKO seedling trays. After 26 days, the plants were transferred into a hydroponic setup. Generic clay balls, net pots, and black polycarbonate tubs were sourced from a local hydroponic shop (Lismore Hydroponics, Lismore, NSW). Five black polypropylene tubs with a volume capacity of 52 L (65 cm (L) × 41 cm (W) x 27 cm (H)) with lids and 30 polypropylene net pots of 250 mL volume (8 cm (D) × 7.5 cm (H) were sourced from the webstore Aqua Gardening. Six holes were drilled in each lid, measuring the collar of the net pots to hold the net pots in place. The net pots were filled with clay balls to hold the plant in place. A total of five hydroponic systems were set up, with each tub containing six plants, representing a replicate (Fig. 1a). The nutrients (3 ml/L water) Coco A and Coco B (CANNA, Netherlands) were added to the tubs, and the water was changed every two weeks. Young leaf tips (Fig. 1b) (harvested every week) and roots (Fig. 1c) were harvested every two weeks from all five replicates to provide the leaf and root samples for the various analyses. Five replicates (one from each hydroponic, ~ 100 mg) of HR and YHL were also collected and immediately snap-frozen in liquid nitrogen for subsequent mRNA extraction.

The HMG were grown by sowing seeds in vermiculite in trays with holes sitting in base trays (Fig. 1d). The seeds were sprouted in the dark for four days in five replicate trays. Once the seedlings emerged, the light cycle was changed to 18 h light and 6 h dark for six days, and the nutrients (2 ml/L water) Coco A and Coco B (CANNA, Netherlands) were added to the base tray for the seedlings to absorb. The six days of light and dark cycle helped to get true leaves and green colour, resulting in ready-to-harvest HMG. After a total of 10 days of growth, 5 small replicate samples were collected and snap-frozen in liquid nitrogen for mRNA extraction. The remaining HMG were also harvested from each replicate by cutting above the roots and were used for subsequent analysis.

Female plants (HAN NE variety) were grown by sowing seeds in HIKOs. The female plants were selected for inflorescence production by determining the sex of plants using the PACE qPCR sex determination method published elsewhere (Toth et al. 2020). Five female plants were potted up and grown under 12 h of light and 12 h of dark cycles.

### Sample analysis

Five independently replicated samples of each tissue were analysed in each case. Where possible, sample analyses were performed by accredited analytical laboratories to obtain certified results. Proximate, nutrients, and heavy metals analysis were provided by the Environmental Analysis Laboratory (EAL), a NATA-accredited laboratory at Southern Cross University (Lismore, NSW, Australia) while cannabinoids (total THC and CBD), terpenoids (profile), caffeine, fatty acid compositions, and vitamins were analyzed by the Therapeutic Goods Authority (TGA) accredited Analytical Research Laboratory (ARL) at Southern Cross University (Lismore, NSW, Australia). Analysis for microbiological pathogens, including *Escherichia coli*, faecal coliforms, and *Salmonella* spp. was conducted by the CP Microbiology and Analytical Lab at Southern Cross University (Lismore, NSW, Australia). The amino acid profiling was performed by the National Measurement Institute (Melbourne, Victoria, Australia).

Full methods used for certified results are included in the supplementary materials (6.1–6.5). Specific details and non-certified protocols are included below.

### Extraction and analysis of lipids

Samples were extracted using 95% hexane. Briefly, the samples were weighed in thimbles, and the weight of each sample was recorded. The thimbles held in the wireframe were placed in pre-weighed extraction flasks, and 130 mL of 95% hexane was added. The samples were refluxed for 1 h and 49 min. Once the cycle was completed, the excess hexane was evaporated, and the flasks were dried in a 105 °C oven for 10 min. Samples were then placed in a desiccator to cool. Once the weight was stabilised, the flasks were weighed to determine the total fat content. The weight of the content was used to calculate the percentage of fat in the plant tissues. The HR were dried in a low-humidity room before extraction because of high moisture content and expected low-fat yield. Once dried, the steps described above were followed.

The extract was then redissolved in 95% hexane and analysed for PUFAs by Gas Chromatography (GC).

Lipid extracts from all the samples were converted to their fatty acid methyl esters (FAMEs) following the method published elsewhere (Ahmad et al. 2019). Fatty acid methyl esters (FAMEs) were analyzed using a gas chromatograph (GC) (Agilent 6890 N, Santa Clara, CA, USA) equipped with an Agilent 6890 flame ionization detector (FID) and a BPX 70 capillary column (70% cyanopropyl polysilphenylene-siloxane, with dimensions 50 m length, 0.22 mm internal diameter, and 0.25 µm film thickness). The FID was maintained at 260 °C, the split injector was set to 230 °C. High-purity helium was used as the carrier gas with a linear flow rate of 1 mL/min. The GC oven temperature was held at 100 °C for 5 min and then increased to 240 °C at a rate of 5 °C/min. Each sample extract (1 μL) was injected with a split ratio of 200:1 and a column flow rate of 1 mL/min. Internal standard methyl tricosanoate at 2 mg/mL solution of BHT (50 mg/L) in hexane was used to calculate the concentration of omega-3, 6, and 9 fatty acids in the extract. For each sample, the area under the curve for each fatty acid was calibrated against the peak area of BHT, adjusted for molecular weight, and then scaled to a concentration of 1 mg per g of extract.

### Total carbohydrate calculation

Total carbohydrate content (% dry weight) was calculated by difference using dry matter content, protein, total fats, and ash content using the following equation:

1 $$\mathrm{Carbohydrate}\;\mathrm{content}\;\left(\%\;\mathrm{DW}\right)\;=\;100\;\mathrm g\;-\;\mathrm{fats}\;\left(\mathrm g/100\;\mathrm g\;\mathrm{DW}\right)\;-\;\mathrm{protein}\;\left(\mathrm g/100\;\mathrm g\;\mathrm{DW}\right)\;-\;\mathrm{ash}\;\left(\mathrm g/100\;\mathrm g\;\mathrm{DW}\right)$$

### Polyphenol extraction and analysis

The concentration of phenolic compounds in the samples was measured as tannic acid equivalent using a spectrophotometer. Phenolic compounds were extracted using 30% ethanol, as 30% has been shown to be the best solvent for extraction (Mailoa et al. 2013). Briefly, dried and ground samples were weighed into 50 ml tubes and ethanol (30% v/v) was added (sample to ethanol ratio 1:10) to each sample and shaken for 24 h. After shaking for 24 h, the samples were centrifuged, and the supernatant was transferred to 10 ml tubes. The samples were diluted before analysis with a spectrophotometer. From each diluted sample, 25 mL samples were transferred to a new 30 mL tube, and 0.5 mL of Folin-Ciocalteu reagent (TanniVer 3 Tannin-Lignin Reagent, HACH, Cat. 256032) and 5 mL of Na_2_CO_3_ solution (HACH, Cat. 67549) were added. Once the reagents were added, the samples were let stand for 25 min. Hydroxylated aromatics, including phenol, tannin, lignin, and cresol, on reaction with the reagent, produce a blue colour, which is proportional to the concentration of these compounds in the sample. Once the reaction was complete, 10 ml of samples was transferred to a cuvette (2 cm) and the concentration was measured using HACH DR 2800 (HACH, Germany) at 700 nm wavelength using the Tannin & Lignin method (Method 8193) stored by the manufacturer.

### RNA extraction, cDNA synthesis and qPCR

Samples were weighed (100 ± 2 mg) into 2 ml Eppendorf tubes and immediately snap-frozen in liquid nitrogen. RNA was extracted using the Qiagen RNeasy Plant Mini Kit following the manufacturer’s instructions. The concentration and purity of RNA samples were determined using a Nanodrop spectrophotometer. The extracted RNA was then subjected to cDNA synthesis using the Qiagen QuantiTect Reverse Transcription Kit following the procedure described by the manufacturer.

Four genes were selected to determine if the cannabinoid synthesis pathway was active in YHL, HMG, and HR. The genes of interest (GI) were olivetolic acid cyclase (OAC), olivetol synthase (OLS), tetrahydrocannabinol acid synthase (THCAS), and cannabidiol acid synthase (CBDAS). Olivetolic acid cyclase and OLS feature early in the cannabinoid synthesis pathway, while THCAS and CBDAS are the genes responsible for the synthesis of the end products. Gene expression primers for each of these genes were published by (Darigh et al. 2023). The expression of OAC, OLS, THCAS, and CBDAS was determined by real-time qPCR. Elongation factor 1 (EF1) and Ubiquitin (UBQ) were used as housekeeping genes using the primers published by (Guo et al. 2018), which were shown to have high amplification efficiencies and specificities for the target genes as well as consistent expression levels over different plant tissues, including root, young plants and leaves. The PCR efficiency of each primer was verified by performing qPCR on serially two-fold diluted samples and generating a six-point standard curve. For each primer pair, the efficiency, regression coefficient, and product Tm were calculated (Supplementary data, Table S1).

qPCR reactions were performed using QIAquant 96 5plex (Qiagen, Germany). Each PCR reaction was 20 µl containing 10 µl of Quantinova SYBR green (Cat. No. 208056, Qiagen, Germany), 1 µl of tenfold diluted cDNA, 0.5 µl of each primer (20 µmol L^−1^), and 8 µl of PCR-grade water. The conditions for the PCR reaction were as follows: activation of polymerase chain reaction at 95ºC for 2 min, 40 amplification cycles at 95 °C for 10 s, 60 °C for 20 s, and melt curve analysis from 60 °C to 95 °C. The data was analysed using Qiagen Q-Rex software.

The expression of genes of interest in YHL, HR and HMG was compared with the expression of genes in inflorescences using the ΔΔCt method. The ∆Ct was calculated for all the samples using EF1 and UBQ as housekeeping genes by subtracting the Ct values of GI from the Ct value of the housekeeping gene of the respective sample. The ∆∆Ct was calculated using inflorescence as a positive control by subtracting the ∆Ct of samples from the ∆Ct of the positive control. The ∆∆Ct was used to calculate relative expression as 2^–∆∆Ct^ for comparison with inflorescences.

### Terpenoid concentration

True concentration of α-pinene was determined for YHL and HMG using the Gas Chromatography (GC) method as previously described (Dimopoulos et al. 2024a). The average concentration of α-pinene was then used to determine the total terpenoid concentration from the total per cent-area of the terpenoid profile.

### Determination of arsenic contamination in clay balls

To determine the source of the arsenic found in the HR, the expanded clay balls were tested for the presence of arsenic. The arsenic extraction was carried out using the method described by (Alam and Tokunaga 2006) with some modifications. Briefly, the clay balls were ground to fine powder in duplicate. The samples were then accurately weighed to 0.40 g in 50 ml polycarbonate centrifuge tubes, and 10 ml of 40% sulfuric acid (v/v) was added to the samples. The samples were shaken overnight using a rotary shaker at room temperature. The extracts were then diluted to bring the final volume to 20 ml and centrifuged at 3500 rpm (Rotofix 32 A, SN 0032620–04, Germany), followed by filtration through Sartorius 0.45 µm cellulose acetate syringe filters. The extracts were then analysed by Inductively Coupled Plasma – Mass Spectrometry (ICP-MS) at EAL, SCU.

### Determination of estimated daily intake (EDI) and nutrient contribution (%NC)

The estimated daily intake (EDI) of calcium, copper, iron, zinc, magnesium, manganese, phosphorus, potassium, zinc, and vitamins was first calculated for YHL and HMG using the measured amount of each nutrient. The USDA reference amount customarily consumed (RACC) was used for both YHL and HMG (85 g) (Eq. 2). The per cent nutrient contribution was then calculated using EDI and the recommended daily intake (RDI) specified for adults by Truswell*, *et al*.* (1990) (Eq. 3). Where the RDI differed between age and sex, the maximum value was selected.

2 $$\mathrm{EDI}\;\left(\mathrm{mg}/\mathrm{day}\right)\;=\;\mathrm{Nutrient}\;\mathrm{content}\;\left(\mathrm{mg}/100\;\mathrm g\;\right)/100\;\times\;85$$

3 $$\mathrm{Nutrient}\;\mathrm{contribution}\;\left(\%\right)\;=\;\mathrm{EDI}/\mathrm{RDI}\;\times100$$

### Statistical analysis

The mean value, standard error (SE), and per cent composition of each analyte have been presented. For each analyte, the statistical analysis was carried out using R version 4.2.2. One-way analysis of variance (ANOVA) (at *p*-value of 0.05) was used to determine if the constitution of analytes varied significantly between the groups using the R stats package (R base package version 4.2.2). Tukey HSD post hoc test was performed to check where the difference occurred between different groups.

## Results

### Macronutrients and proximate

The moisture content (± SE) was 81.8% (± 0.9), 87.8% (± 3.5), and 88.76% (± 0.8) for YHL, HR, and HMG, respectively (based on drying at 105 °C). Table 1 compares the ash content (after burning volatile matter at 550 °C), crude protein (calculated from total nitrogen), total fat, and total carbohydrate content in YHL, HR, and HMG of hemp.

**Table 1:** Moisture, ash and macronutrient composition, including crude protein, total carbohydrates, and fat content (with concentration of PUFAs) in YHL, HR, and HMG. Fat content (dry weight basis) for YHL and HMG was calculated from fresh weight content using the moisture content, and vice versa for HR. < indicates below the limit of detection as given. Different superscript letters signify significant difference (at *p* ≤ 0.05, *n* = 5) across different tissues within the same row (Tukey’s HSD)

Analyte/Tissues	YHL	HMG	HR
Moisture FW	81.81 ± 0.9^a^	88.76 ± 0.08^a^	87.80 ± 3.5^a^
Ash content (% DW ± SE)	13.98 ± 0.2^a^	10.62 ± 0.2^b^	10.31 ± 0.9^b^
Crude Protein (% DW ± SE)	46.04 ± 0.2^a^	42.3 ± 0.4^a^	24.14 ± 2.3^b^
Total carbohydrates (% DW ± SE)	12.62 ± 1.3^a^	27.90 ± 0.8^c^	57.92 ± 2.9^b^
Total Fat in mg/g DW (± SE)	27.35 ± 1.0^a^	19.17 ± 0.8^c^	7.63 ± 0.2^b^
Total Fat in mg/g FW (± SE)	5.11 ± 0.2^a^	2.16 ± 0.1^c^	1.04 ± 0.02^b^
ω−3 fatty acids (% total fat ± SE)	15.88 ± 3.4^a^	19.75 ± 0.9^a^	1.14 ± 0.1^b^
ω−6 fatty acids (% total fat ± SE)	1.62 ± 0.2^a^	4.5 ± 0.4^b^	1.94 ± 0.3^a^
ω−7 fatty acids (% total fat ± SE)	0.26 ± 0.04^a^	0.78 ± 0.1^c^	< 0.1^b^
ω−9 fatty acids (% total fat ± SE)	0.56 ± 0.1^a^	1.76 ± 0.7^a^	0.68 ± 0.2^a^

The average crude protein in HR was significantly lower (*p* ≤ 0.05) compared to HMG and YHL at 57% and 52%, respectively, which were not different from each other (Table 1). The ash content was significantly higher in YHL when compared with HMG and HR (*p* ≤ 0.05); however, no significant difference was observed between HR and HMG. The average carbohydrate content ranged from 12.62% in YHL to 57.92% in HR. The total fat content was higher in YHL, followed by HMG, while HR had the lowest concentration of total fat (Table 1).

The concentration of PUFAs, including ω−3, 6, 7, and 9 fatty acids, was higher in HMG as compared to YHL and HR (Table 1). ω−7 was not detected in HR, with only very low concentrations of ω−3, 6, and 9 fatty acids detected in this tissue. The ω−3 fatty acids made up 15.88% of total fats in YHL, while the concentration of ω−6, 7 and 9 fatty acids was much lower at 1.62%, 0.26%, and 0.56%, respectively (Table 1).

### Amino acids

Table 2 summarises the concentration of amino acids in hemp YHL, HMG and HR. Out of 21 major amino acids, 17 were detected in hemp tissues, with YHL containing the highest quantities. Glutamic acid was present with the highest concentration in all the tissues, followed by aspartic acid, while the concentration of taurine was 0.05 mg/g of freeze-dried weight of tissues. The concentration of all the other amino acids was significantly higher in YHL compared to HMG and HR, except for aspartic acid, glutamic acid, and histidine, where no significant difference was observed between YHL and HMG. Hydroxyproline was the only amino acid with a higher concentration in HR than YHL and HMG. Out of nine essential amino acids (EAAs), histidine, threonine, valine, lysine, isoleucine, leucine, phenylalanine, and methionine were present in significant concentration, with the highest concentration in YHL, followed by HMG, while HR had the least amount.

**Table 2:** Average concentration of amino acids in YHL, HMG and HR of hemp. The concentration (mg/g ± SE) given is based on the dry weight of each tissue. < indicates below the limit of detection as given. Different superscript letters signify significant difference (at *p* ≤ 0.05, *n* = 5) across different tissues within the same row (Tukey’s HSD)

Amino acid/Tissue	YHL	HMG	HR
Aspartic Acid	36.2 ± 1.2^a^	36.8 ± 1.39^a^	11.78 ± 1.29^b^
Serine	18 ± 0.32^a^	14.4 ± 0.40^b^	8.48 ± 0.84^c^
Glutamic Acid	42 ± 1.18^a^	39.2 ± 1.59^a^	12.45 ± 1.47^b^
Glycine	15.4 ± 0.4^a^	10.8 ± 0.49^b^	5.15 ± 0.53^c^
Histidine	5.86 ± 0.19^a^	5.48 ± 0.33^a^	1.83 ± 0.22^b^
Arginine	22 ± 0.32^a^	18.8 ± 0.37^b^	5.05 ± 0.67^c^
Threonine	17 ± 0.32^a^	12.4 ± 0.24^b^	5.5 ± 0.53^c^
Alanine	18.8 ± 0.49^a^	15.6 ± 0.40^b^	6.73 ± 0.66^c^
Proline	15.2 ± 0.20^a^	12.56 ± 0.72^b^	5.05 ± 0.53^c^
Tyrosine	11.8 ± 0.20^a^	7.84 ± 0.12^b^	3.33 ± 0.31^c^
Valine	16.6 ± 0.24^a^	12 ± 0.32^b^	5.18 ± 0.57^c^
Lysine	25.2 ± 0.58^a^	16.8 ± 0.73^b^	9.13 ± 1.10^c^
Isoleucine	12.6 ± 0.24^a^	8.68 ± 0.16^b^	3.48 ± 0.49^c^
Leucine	27 ± 0.71^a^	19.4 ± 0.51^b^	7.65 ± 0.96^c^
Phenylalanine	15.6 ± 0.24^a^	10.8 ± 0.37^b^	4.35 ± 0.50^c^
Methionine	3.78 ± 0.05^a^	2.11 ± 0.29^b^	0.84 ± 0.13^c^
Hydroxyproline	0.91 ± 0.0^a^	1.46 ± 0.09^b^	6.35 ± 0.16^c^
Taurine	< 0.05	< 0.05	< 0.05
Total amino acids	303.95^a^ ± 6.91	245.13^b^ ± 8.54	102.29^c^ ± 10.94
Total essential amino acids	123.64^a^ ± 2.58	87.67^b^ ± 2.96	37.94^c^ ± 4.94

### Micronutrients

#### Minerals

Table 3 provides a summary of micronutrients analysed in YHL, HR and HMG, where the concentration is given in mg/g based on the dry weight of the samples. Potassium content was highest in YHL (40.14 mg/g), followed by HMG (26.72 mg/g) and HR (14.98 mg/g). Average Phosphorus content in YHL, HR and HMG was 13.76 mg/g, 12.56 mg/g, and 13.54 mg/g, respectively, with no significant difference observed between tissues (*p* < 0.05). Silicon content varied significantly between different tissues (*p* < 0.05), with the highest concentration in YHL (2.98 mg/g) and the lowest concentration in HMG (0.64 mg/g), and the concentration in HR was 1.10 mg/g. YHL had a higher concentration of calcium (27.26 mg/g) as compared to HR (6.54 mg/g) and HMG (12.42 mg/g), while HR had a high concentration of sodium (12.68 mg/g) and copper (0.05 mg/g). Fe content in HMG was more than two times higher than in YHL and HR. Boron, magnesium, and zinc were also present in low concentrations and varied between the tissues. The concentration of molybdenum was very low as compared to other minerals and varied significantly between YHL, HR, and HMG (Table 3).

**Table 3:** Average concentration (mg/g ± SE, *n* = 5) of inorganic micronutrients in YHL, HR and HMG of hemp. The concentration given is based on the dry weight of each tissue. < indicates below the limit of detection as given. Different superscript letters signify significant difference (at *p* ≤ 0.05) across different tissues within the same row (Tukey’s HSD)

Analyte/Tissues	YHL	HMG	HR
Iron	0.12 ± 0.002 ^a^	0.27 ± 0.02^b^	0.13 ± 0.012^a^
Magnesium	8.36 ± 0.48^ab^	7.9 ± 0.18^a^	11.84 ± 1.62^b^
Zinc	0.06 ± 0.003^a^	0.07 ± 0.00^a^	0.04 ± 0.004^b^
Calcium	27.26 ± 01.17^a^	12.4 ± 0.60^c^	6.54 ± 0.47^b^
Sodium	< 0.1	0.20 ± 0.00^b^	12.68 ± 2.63^a^
Phosphorus	13.76 ± 0.47^a^	13.54 ± 0.33^a^	12.56 ± 2.02^a^
Potassium	40.14 ± 1.85^a^	26.72 ± 0.67^c^	14.98 ± 3.67^b^
Copper	0.01 ± 0.001^a^	0.02 ± 0.002^a^	0.05 ± 0.003^b^
Manganese	0.04 ± 0.003^a^	0.08 ± 0.002^c^	0.02 ± 0.002^b^
Boron	0.05 ± 0.002^a^	0.04 ± 0.00^c^	0.02 ± 0.00^b^
Molybdenum	0.007 ± 0.001^a^	0.002 ± 0.0002^c^	0.01 ± 0.003^b^
Silicon	2.98 ± 0.16^a^	0.64 ± 0.07^c^	1.1 ± 0.04^b^

#### Vitamins

Vitamin C was not detected in YHL, HR or HMG samples. The content of vitamin E (α-tocopherol) was higher in HMG (11.04 mg/g) than in YHL, where the content was 73.4% lower compared to HMG. Vitamin B1 (thiamine) was significantly higher in YHL compared to HR and HMG. Vitamin B2 (riboflavin) was present in YHL and HMG, whereas it was not found in root samples. Vitamin B3 (nicotinamide) was only detected in YHL, while B3 (nicotinic acid) was detected in all the tissues with no significant difference between the tissues (Table 4).

**Table 4:** Vitamin content (mg/g ± SE) in YHL, HR, and HMG based on freeze-dried weight (FDW). Results expressed as mean ± standard error (*n* = 5); different superscript letters signify significant difference (at *p* ≤ 0.05) across different tissues within the same row (Tukey’s HSD)

Analyte/Tissues		YHL	HMG	HR
Vitamin E (α-tocopherol)		2.94 ± 0.53^a^	11.04 ± 2.39^b^	< 0.001
Vitamin C (Ascorbic acid)		<0.001	<0.001	< 0.001
B Vitamins	B1 (Thiamine)	2.66 ± 0.19^a^	0.48 ± 0.27^b^	0.81 ± 0.49^b^
	B2 (Riboflavin)	0.03 ± 0.006^a^	0.03 ± 0.006^a^	< 0.001
	B3 (Nicotinamide)	0.08 ± 0.04	< 0.001	< 0.001
	B3 (Nicotinic acid)	3.09 ± 0.48^a^	2.71 ± 0.49^a^	2.97 ± 0.54^a^
	B9 (Folate)	< 0.001	< 0.001	< 0.001

### Recommended daily intake, estimated daily intake and nutrient contribution

Based on the concentration of micronutrients and EAAs detected, their RDIs, EDIs and NC were calculated (Table 5).

**Table 5:** Recommended daily intake of some essential nutrients and EAAs for adults (Truswell et al. 1990) and calculated estimated intake (EDI) and nutrient contribution (NC) based on the serving size of 85 g

Nutrient	RDI (mg)	YHL		HMG	
		EDI (mg)	NC (%)	EDI (mg)	NC (%)
Calcium	1000	421.47	42.15	118.67	11.87
Copper	1.7	0.21	12.35	0.17	10.00
Iron	18	1.82	10.11	2.59	14.39
Magnesium	420	129.25	30.77	75.67	18.02
Manganese	5.5	0.67	12.18	0.8	14.55
Molybdenum	0.045	0.11	244.44	0.022	48.89
Phosphorus	1000	212.75	21.28	129.36	12.94
Potassium	3800	620.62	16.33	255.28	6.72
Zinc	14	0.98	7.00	0.71	5.07
Vitamin B1 (Thiamine)	1.2	41.06	3421.67	4.6	383.33
Vitamin B2 (Riboflavin)	1.3	0.43	33.08	0.26	20.00
Vitamin B3 (Niacin)	16	47.83	298.94	25.91	161.94
Vitamin E (α-Tocopherol)	10	45.45	454.50	105.47	1054.70
Histidine	700	90.60	12.94	52.36	7.48
Threonine	1050	262.85	25.03	118.47	11.28
Valine	1820	256.66	14.10	114.65	6.30
Lysine	2100	389.63	18.55	160.51	7.64
Isoleucine	1400	194.81	13.92	82.93	5.92
Leucine	2730	417.46	15.29	185.35	6.79
Phenylalanine + Tyrosine	1750	424.15	24.24	177.65	10.15
Methionine	1050	58.44	5.57	20.16	1.92

### Heavy metals

The accumulation of heavy metals, lead, mercury, chromium, and arsenic in YHL and HMG was lower than the detection limits, which were < 0.5 mg/kg, < 0.1 mg/kg, < 1 mg/kg, and < 0.1 mg/kg, respectively (Table 6). The concentration of lead and chromium was lower than the detection limit in the HR, while the concentration of mercury was 0.12 (Table 6). The accumulation of arsenic was observed in HR with an average concentration of 1.38 mg/kg (Table 6). The expanded clay balls used in the hydroponic system revealed the presence of arsenic at a concentration of 5 ppm.

**Table 6:** The concentration of heavy metals in the YHL, HR, and HMG of hemp. The concentration is given as the mean ± SE (mg/kg) and is based on the dry weight

Analyte	Tissues		
	YHL	HMG	HR
Lead	< 0.5	< 0.5	< 0.5
Mercury	< 0.1	< 0.1	0.12 ± 0.00
Chromium	< 1	< 1	< 1
Arsenic	< 0.1	< 0.1	1.38 ± 0.3

### Phenolic compounds and caffeine

The concentration of tannins/polyphenols measured as tannic acid was significantly different between the tissues (*p* < 0.05), with the highest in HMG (7.42 mg/g), followed by YHL (5.56 mg/g) and HR (2.18 mg/g). No caffeine was detected in YHL, HMG, or HR.

### Microbiological pathogens

The microbiological results showed *E. coli* and fecal coliforms were < 3 MPN/g of fresh YHL, HR, and HMG for each of the five replicates. *Salmonella* spp. was not detected (based on 25 g of fresh weight) in any tissue samples.

### Gene expression

**Fig. 2:**
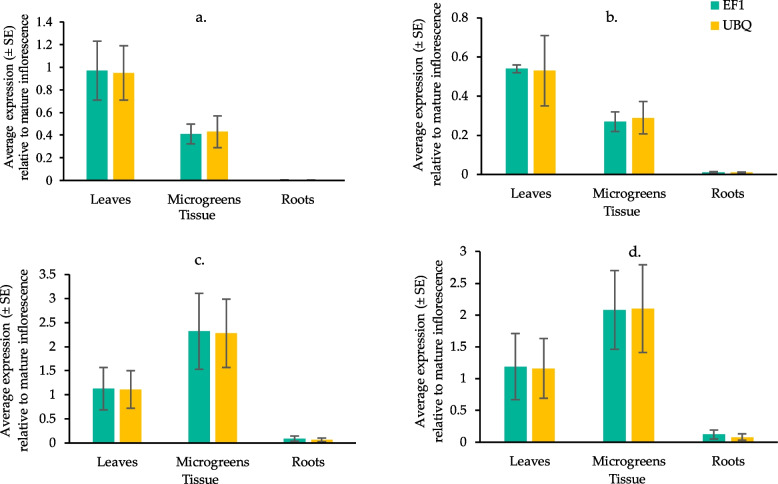
Rt-qPCR of OAC (**a**), OLS (**b**), THCAS (**c**), and CBDAS (**d**) in YHL, HMG, and HR of *Cannabis sativa* L. mRNA expression (± SE, *n* = 5) is relative to the expression of respective gene in mature female inflorescences calculated as 2^-ddCq^ using EF1 or UBQ as reference genes. The legend shows the two reference genes used to calculate the respective relative expressions

The expression of OLS in YHL was about half of that of the inflorescence, while the expression of OAC, THCAS, and CBDAS was similar to the inflorescence. A similar pattern was observed when using either reference gene for the calculations (Fig. 2).

Compared to inflorescences, the expression of all target genes was very low in HR, with OAC and OLS below 0.01, while THCAS and CBDAS were below 0.1 when averaging over the two housekeeping genes (Fig. 2). A much different trend was observed in the expression of genes in HMG, where cannabinoid synthase genes THCAS and CBDAS were expressed about two times as high in mature inflorescences. In contrast, the expression of OAC and OLS was less than 0.5 times the expression in inflorescences (Fig. 2).

### Terpenoid profile

**Fig. 3:**
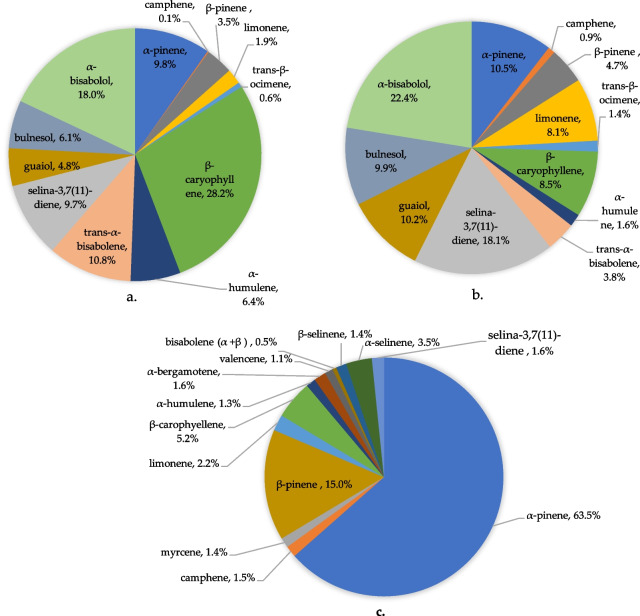
Profile of major compounds detected in YHL (**a**) and HMG (**b**) compared with the terpene profile of HAN NE inflorescence (**c**) (Dimopoulos et al. 2024b). The Area% gives the abundance of respective compounds in relation to the total peak area of all the compounds detected

The comparative analysis of terpenoid profiles in YHL (Fig. 3a) and HMG (Fig. 3b) revealed notable differences in their chemical composition, and is compared with the terpenoid profile of hemp inflorescence (Fig. 3c). In YHL, β-caryophyllene emerges as the most abundant terpenoid, accounting for 28.2% of the total content, followed by α-bisabolol (18%), α-pinene (9.8%) and selina-3,7(11)-diene (8.9%). In contrast, microgreens show a marked shift in terpenoid composition, with α-bisabolol (22.4%) and selina-3,7(11)-diene (18.1%) becoming the predominant constituents. While α-bisabolol remains consistently present in both tissues, β-caryophyllene, the most common terpene in hemp (Ninkuu et al. 2021), levels drop sharply to just 8.5% in microgreens compared to 28.2% in young leaves.

### Cannabinoid and terpenoid content

Overall, cannabinoid and terpenoid concentrations were low (< 0.02% FW for cannabinoids and < 0.1% FW for terpenoids). The CBD in HMG was significantly higher (*p* < 0.05) than in YHL, while no CBD was detected in HR. The THC content was significantly higher in YHL as compared to HR and HMG (*p* < 0.05). CBD was clearly the dominant cannabinoid in HMG, whereas for YHL, THC and CBD concentrations were at similar levels (*p* > 0.05) (Table 7). The terpenoid content was notably higher in YHL in comparison to HMG, while no terpenes were detected in HR (Table 7).

**Table 7:** The concentration of total CBD (CBD + CBDA) and THC (THC + THCA) is given in mg/g (± SE) on fresh weight (FW) and dry weight basis (DW calculated from fresh weight using average moisture content) basis for YHL, HR, and HMG determined using gas chromatography. Total terpenoid concentration is given in mg/g calculated using the true concentration of α-pinene and total peak area. Different superscript letters signify significant difference (at *p* ≤ 0.05) across different tissues within the same row (Tukey’s HSD)

Analyte/Tissues	YHL	HMG	HR
CBD FW	0.06 ± 0.02^a^	0.12 ± 0.02^b^	< 0.002
CBD DW	0.32 ± 0.08^a^	1.08 ± 0.14^b^	< 0.02
THC FW	0.08 ± 0.01^a^	0.02 ± 0.002^b^	< 0.002
THC DW	0.45 ± 0.04^a^	0.14 ± 0.02^b^	< 0.02
Total terpenoid concentration (calculated FW)	0.92 ± 0.16^a^	0.06 ± 0.002^b^	< 0.01

## Discussion

### YHL and HMG have high value for human nutrition

The YHL and HMG contained a similar content of crude protein, which was higher than is known for various other leafy vegetables such as lettuce, spinach, and coriander (Singh et al. 2001), whereas the concentration of unsaturated fatty acid was high but lower than has been found in spinach (Barzegar et al. 2007). The carbohydrate content in YHL and HMG was lower when compared to spinach and kale (Agarwal et al. 2017). The combination of low carbohydrate content, low fat content highlighted the potential of these hemp tissues as health-promoting foods. Consumption of plant-based protein is known to increase the intake of various co-nutrients such as potassium, phosphorus, magnesium, and folic acid (Ahnen et al. 2019), while consumption of plant protein has been claimed to reduce cardiovascular diseases, low-density lipoprotein (LDL), obesity, and type II diabetes (Sá et al. 2020; Nurkolis et al. 2025). Replacing saturated fatty acids (SFAs) with polyunsaturated fatty acids (PUFAs) has been associated with reduced risk of cardiovascular diseases (Sacks et al. 2017) and intake of foods with low ω−6 to ω−3 ratios, such as found here for YHL and especially HMG, has been linked with reduced risk of chronic diseases (Innis 2014). In terms of amino acids, EAAs were present in considerable concentrations in HMG and especially YHL (about twice as high in YHL compared to HMG on average) and were similar in abundances as has been reported for other leafy greens such as spinach (Lisiewska et al. 2011) and kale (Lisiewska et al. 2011, 2008), thus these hemp tissues are likely to be good source of EAAs assuming a similar bioavailability as for other leafy greens.

As evident from Tables 2, 3, 4, 5, HMG and YHL have the potential to contribute significantly to the recommended daily intake of micronutrients. Overall, the YHL produced in this study contained more micronutrients than the HMG, with the notable exception of iron and α-tocopherol. The finding for α-tocopherol, which is the most biologically active form of vitamin E with in vivo antioxidant properties, was similar to what was observed by (Ghoora et al. 2020) who compared the content of α-tocopherol in HMG and mature leaves of fenugreek, spinach, and roselle. The values for the HMG were overall in congruence with (Corrado et al. 2022) who analysed microgreens of six different varieties of hemp. Calcium, magnesium, phosphorus, and iron were present in similar or higher quantities in HMG when compared to the nutritional content of microgreens of radish, spinach, carrot, fennel, fenugreek, onion, roselle and sunflower (Ghoora et al. 2020). The content of α-tocopherol in HMG (1.24 mg/g (FW)) was higher compared to popular microgreens with this data available, including radish, spinach, carrot, fennel, fenugreek, onion, roselle and sunflower. When compared to romaine lettuce and crisphead lettuce (Kim et al. 2016a), the concentration of zinc, iron, calcium, phosphorus, magnesium, manganese, and potassium was found to be higher in YHL, indicating the potential of YHL as a nutritional salad. Based on EDI, YHL can contribute 42%, 31%, 21%, and 16% to the daily recommended amount of calcium, magnesium, phosphorus, and potassium, respectively (Table 5). HMG and YHL can also be a good source of vitamin B3 (nicotinic acid), while vitamin B2 (riboflavin) was also present in low amounts in YHL and HMG. YHL also had a good amount of Vitamin B1 (thiamine). Both YHL and YHL can thus play an important role as a source of various micronutrients to prevent or address deficiencies in iron and calcium, in particular. While YHL were generally higher in nutritional value, HMG, on the other hand, had a higher iron content. When compared to spinach (Barzegar et al. 2007), the iron content was lower in YHL; however, iron content was 60% higher in HMG compared to spinach microgreens (Ghoora et al. 2020). Iron content in YHL, however, when compared to some common leafy salads such as lettuce and spinach, was lower (Barzegar et al. 2007; Kim et al. 2016b). Calcium content in YHL, on the other hand, was nearly five times higher than in spinach (Barzegar et al. 2007), while the calcium content of HMG was seven times higher compared to spinach microgreens (Ghoora et al. 2020).

### HR as a tea ingredient or coffee substitute

HR were higher in total carbohydrates and lower in protein and fats compared to YHL and HMG. No cannabinoids or terpenoids were detected in HR, which matched the very low expression levels of the various genes of the biosynthesis pathway. Furthermore, as HR are not intended to be consumed fresh as salad, as is the case for the YHL and HMG, but rather via herbal tea or coffee substitute after roasting, the EDI and NC for the various amino acids and micronutrients were not calculated. According to our results, HR may provide benefits due to the presence of water-soluble vitamin B3. Vitamin B3 has been shown to have various health-related benefits, including antioxidant, antidiabetic, and neurological benefits (Lisiewska et al. 2011, 2008; Ghoora et al. 2020).

Surprisingly, in this study, arsenic and mercury accumulation was found in HR at an average level of 1.38 mg/kg and 0.12 mg/kg (DW), respectively (Table 6). The arsenic concentration was above the limits (1 mg/kg) set by FSANZ for food such as rice, seaweed, molluscs, and fish; however, the concentration of mercury was lower than the maximum permissible limit (0.5 mg/kg) in seafoods. Exposure to arsenic at toxic levels has been associated with an increased risk of diverse health concerns, including reproductive, neural, cardiovascular, dermal, renal, hepatic, and physiological (Ozturk et al. 2022). Notably, the concentration of arsenic was relatively high in HR, but the concentration in YHL of the same plants was below the detection limit (< 0.1 mg/kg). Previous studies have also shown a significantly higher accumulation of As in roots than in leaves and stems (Picchi et al. 2022). While arsenic accumulation is indicative of a high potential for the use of hemp in phytoremediation (Linger et al. 2002), it also indicates that extra care needs to be employed when producing HR for human nutrition, such as ensuring the water/medium used has negligible levels of heavy metals and periodical testing for these in the product. The most likely explanation of arsenic contamination in this study is the clay balls used in the netted pots of the hydroponics setup, which, on testing, were found to contain 5 ppm arsenic.

As the HR produced in this study showed As contamination and analyses on water extracts were beyond the scope of this study, the potential value of HR for human nutrition in the form of herbal extract, or coffee substitute, needs further study.

### YHL and HMG have relatively high polyphenol levels

Tannins and lignin, together with cresols and phenols analysed as tannic acid, were highest in YHL, followed by HMG and HR. When compared to spinach and silverbeet, YHL and HMG contained higher amounts of tannin (Sotelo et al. 2010). These amounts were much lower, however, than normally present in non-herbal teas and coffee (Savolainen 1992). Tannins have been linked with various anti-nutritional properties because of their ability to form complexes with proteins and enzymes and their ability to reduce the bioavailability of iron and other minerals (Sahakyan et al. 2020). If the presence of tannins and other polyphenols in hemp tissues affects the bioavailability of nutrients, this will need to be addressed, such as by breeding to reduce the phenolics in the plant. However, health-promoting properties of tannins have also been well documented; for instance, tannic acid has been shown to have anticancer properties in various rodent models by either oral administration of green tea or by topical application (Chung et al. 1998). Further, tannins have anti-inflammatory, antimutagenic, antioxidant, antidiabetic, and cardioprotective properties (Kumari and Jain 2012). Enrichment of foods with tannins has been shown to promote healthy changes in gut microbiota (Molino et al. 2021) Thus, YHL, as it contains phenolic compounds, can be used as a health-promoting green tea with various benefits while containing no caffeine content.

### YHL and HMG can produce cannabinoids and terpenoids at low levels

It is well-established that cannabinoids are produced in the glandular trichomes, which mainly form in female inflorescences but can also be found on leaves and stems outside the inflorescence (Mahlberg and Kim 2004). In agreement with this, the results of this study showed that the genes involved in cannabinoid biosynthesis were active in vegetative YHL and HMG but inactive in HR, which was further corroborated by the cannabinoid concentrations found in these tissues. As we used GC-FID for cannabinoid quantification, which converts THCA and CBDA into THC and CBD during analyses, we can only report on total THC (THC + THCA) and total CBD (CBD + CBDA) levels. This method is often preferred for regulatory testing as it provides the drug potential of a substance. Why the total CBD concentration was higher than total THC in HMG (0.012% and 0.002% w/w FW, respectively), whereas no significant difference was observed between total THC and total CBD content of YHL (0.006% and 0.008% w/w FW, respectively) remains unclear. The presence of cannabinoids can be an obstacle when proposing the use of YHL and HMG as food due to the expected regulatory requirements for these foods. FSANZ regulation (Code et al. 2025) allows a maximum limit of 0.0075% of CBD for currently approved dehulled seeds and hemp seed oil for human consumption, which is exceeded by the concentration of total CBD in HMG, measured at 0.012% FW. The THC limits are stricter again, allowing maximum limits of 0.0005%, 0.001%, and 0.00002% for dehulled hemp seeds, hemp seed oil and beverages prepared from low THC seeds, respectively. Total THC concentration in HMG and YHL (0.002% and 0.008% w/w FW, respectively) thus also exceeded the prescribed limit of currently approved products. The higher concentration of cannabinoids in these tissues appears problematic in regard to the FSANZ regulation, as it exists for other hemp food sources, which will need to be addressed. Other studies have reported lower concentrations in HMG (CBD content in Finola, Antal, Silvana, Kompolti, Uso31, and Tisza HMG varied from 0.001% to 0.0023% while the THC content in the HMG ranged from 0.00005% to 0.0001% on fresh weight basis between varieties (Pannico et al. 2022)) indicating that variety selection is an important factor for controlling cannabinoid content. Another consideration is that, especially for YHL and HMG to be used as fresh salads, the large majority of the cannabinoids will be in the acid form, which is not psychoactive when taken orally (Small 2015).

While similar levels of cannabinoids were present in YHL and HMG, the terpenoid profile of HMG and YL varied noticeably from each other and the mature inflorescence. While the inflorescence was dominated by α-pinene, most of the terpenoids detected in YHL and HMG were sesquiterpenes. This can be due to the type of trichomes on the tissues (sessile or stalked trichomes) as sessile trichomes are known to be more abundant on young tissues, and the type of trichomes have been shown to affect the type of terpenes synthesised (Livingston et al. 2020). Terpenes, including those found in *Cannabis*, have been shown to have various therapeutic effects and are widely used as an additive to foods and drinks to give them fragrance. Studies have shown various biologically important properties associated with β-caryophyllene, α-bisabolol, and α-pinene, including against inflammation, cancer, oxidative stress, and nervous system diseases (Scandiffio et al. 2020; Francomano et al. 2019; Machado et al. 2018; Eddin et al. 2022; Rocha et al. 2011; Özbek and SEVER YILMAZ, B. 2017). As there were no terpenes found in the root and only low concentrations in HMG and YHL, it’s unlikely that there are any health issues with consuming these tissues in relation to terpenoids and cannabinoids, and neither are health-promoting effects likely.

### Considerations on producing YHL, HMG and HR in a commercial setting

In the hydroponic setup utilised in this study, fresh YHL could be harvested as frequently as every week, yielding approximately 200 g FW each setup. HR were harvested once every three weeks, yielding 500 g FW. After harvesting all the free HR (retaining the roots in net pots), which was combined with trimming of the shoots, the plants successfully recovered so that they could be used again for producing more YHL and HR. This method could be adapted and scaled up to an industrial setting (with further improvements where required), providing continuous production of YHL and HR from the same setup for efficient production. The successful production of HMG by germinating seeds in beds of vermiculite and using a 10-day growth period was adopted after sprouting seeds in a commercial seed sprouter failed due to contamination issues. HMG production was efficient but more laborious than YHL or HR production, as each batch needed to be started from scratch. As no faecal coliforms, *E. coli* or *Salmonella spp.* were detected in the samples, the clean production environment used in this study met the safety guidelines for food production set by FSANZ (Dimopoulos et al. 2024a).

## Conclusions

The use of vegetative hemp tissues, such as YHL, HR, and HMG, for human consumption shows promise for promoting human health and as a way of diversifying and adding value to the industrial hemp industry. YHL and YHL were determined to be high in various proteins, EAAs and PUFAs while having low carbohydrate and saturated fat content, indicative of a healthy food. They also have the potential to play an important role in meeting the RDI for various nutrients, including iron, calcium, silicon, vitamin B1, vitamin B3 and vitamin E. The cannabinoid biosynthesis pathway was found to be active in HMG and YHL but not in HR, which was in agreement with the presence of cannabinoids in YHL and HMG and the absence of cannabinoids in HR. Only low levels of cannabinoids and terpenoids were detected in YHL and HMG which are unlikely to have any therapeutic or harmful effects, also considering that cannabinoids will be predominantly in their acidic forms in fresh tissues. While low, the CBD and THC levels detected (by GC-FID) in YHL and HMG in this study were higher than the current limits set by FSANZ for approved hemp foods (seeds and seed-derived products), highlighting the importance of selecting appropriate varieties. Accumulation of arsenic in HR was observed, indicating that safely cultivating HR for food requires standardised procedures and would benefit from monitoring for toxins. Further studies are required to evaluate derivative products, such as hemp tea, hemp leaf herbal extract, and hemp root coffee, to determine their nutritional values and health benefits.

## Supplementary Information

Supplementary Material 1

## Data Availability

The authors declare that the data supporting the findings of this study are available within the paper and its Supplementary Information. Further data supporting the findings of this study can be obtained from the corresponding author, upon reasonable request.
