# Supplementary material for: The potential of hemp roots, microgreens and leaves for human nutrition
Source: J Cannabis Res. 2025 Oct 7;7:75. doi: 10.1186/s42238-025-00319-2 (PMC12502590; doi:10.1186/s42238-025-00319-2)
Supplement: Supplementary file 1 — Supplementary Material 1 [file 42238_2025_319_MOESM1_ESM.docx]

**Supplementary Materials:**

**Table S1**. Sequence, PCR efficiency, Tm, and coefficient of determination (R^2^) of primers used in qPCR to determine the expression of key cannabinoid pathway genes.

| **Gene** | **Primer Sequence (5' - 3')** | | **Amplicon length (bp)** | **Tm (℃)** | **PCR**  **efficiency** | **Coefficient of determination (R^2^)** |
| --- | --- | --- | --- | --- | --- | --- |
|  | **Forward** | **Reverse** |  |  |  |  |
| EF1⍺ | TGTTTTGCACGGATCAGTTTG | AATGCCGACCGCTACAGTTC | 100 | 59.3 | 1.01 | 0.97 |
| UBQ | TACTGCGCCAGCTAACAAACC | GCACCCGTCTGACCTGAATC | 115 | 60.6 | 1.06 | 0.98 |
| OAC | AAAGATGAAATCACAGAAGCCCAA | TGAGTCACATCTTTACCCCAGT | 110 | 61 | 1.01 | 0.97 |
| OLS | ACATAATGGCTTGCTTGTTTCGT | GATTGTTTGCCCAGTTGACAC | 167 | 59.7 | 0.99 | 0.98 |
| CBDAS | CTTTAGTGGAGGAGGCTATGGA | CACCACCACGTATAGCCCA | 149 | 58.7 | 1 | 0.99 |
| THCAS | CGTGGGTTGAAGCCGGAG | CCATAGCCTCCTCCACTAAAGT | 133 | 59.1 | 0.99 | 0.99 |

6.1 Protein Analysis by LECO TruMac CNS Analyser

Crude protein was calculated by determining the total nitrogen content in the samples by LECO (LECO, Michigan, USA). Samples were dried at 40°C for 24 hours and ground to <0.5 mm using a ring mill grinder. Approximately 0.2 g of dried and ground sample was weighed into the ceramic sample boats, and the weight was recorded. The ceramic sample boats were loaded into the LECO TruMac CNS analyser (Model 630-300-200). The samples were analysed following the operating instructions detailed in the LECO TruMac Operation Manual. A seven-point calibration curve was generated using sulfamethazin standard (LECO, Cat. no. 502-694, Lot no. 1003) for 0.5, 1, 2, 3 mg elemental nitrogen concentration and sulfamethazin standard (LECO, Cat. no. 502-657, Lot no. 1002) for 20, 30, and 40 mg elemental nitrogen concentration. The concentration of nitrogen in samples was calculated by the instrument using the linear regression formula generated from the standard calibration curve.

6.2 Micronutrients and heavy metals analysis

For micronutrients and heavy metal analysis, acid digestion was carried out by heating the sample in concentrated nitric acid using a Teflon®-coated graphite HotBlock. Briefly, approximately 0.4g of plant samples were weighed into 50ml disposable digestion tubes (SC475) and the actual weight was recorded for final calculations. To the samples, 10 mL nitric acid (98%) was added, and tubes were placed into a preheated (120 °C) HotBlock digestor. The samples were digested for one hour. Following digestion, the samples were cooled down, and the volume was made to 25 mL with Milli-Q water. After allowing the samples to settle for approximately two hours, the samples were transferred to 10 mL sample tubes for reading on ICPMS. Root and leaf samples were analysed using ICPMS NexION 2000B, and microgreen samples were analysed using ICPMS NexION 350D. The instruments were operated following the manufacturer's instructions. The operational conditions for both instruments are given below:

**Instrument: ICP MS NexION 2000B**

**Background vacuum (Plasma off): <1.0 x 10^-8^**

**Operating Parameters (30mins + Plasma on)**

Operating Vacuum (Low): 2.6 x 10^-7^

Coolant Temperature (^0^C): 21

Interface Temperature (^0^C): 37

Torch Box Temperature (^0^C): 56

Net back pressure (psi): 65

Neb gas flow (L/min): 0.86

Plasma RF Power (W): 1600

**Sample Intro Components**

Neb Type: PFA

Spray chamber type: Quartz

**Instrument: ICP MS NexION 350D**

**Background vacuum (Plasma off): <1.0 x 10^-8^**

**Operating Parameters (30mins + Plasma on)**

Operating Vacuum (Low): 7 x 10^-8^

Coolant Temperature (^0^C): 21

Interface Temperature (^0^C): 38

Torch Box Temperature (^0^C): 45

Net back pressure (psi): 41

Neb gas flow (L/min): 1.05

Plasma RF Power (W): 1500

**Sample Intro Components**

Neb Type: PFA

Spray chamber type: PC3

6.3 Determination of cannabinoids and terpenoids

Cannabinoid concentration and terpenoid composition were determined using Gas Chromatography with Flame Ionisation Detector (GC-FID). Briefly, 1g of raw hemp samples were weighed into a 50 mL Falcon tubes. Methanol (30mL) was added to the samples and sonicated, followed by centrifugation at 3000rpm for five minutes. The supernatant was transferred to 50mL volumetric flasks. A second extraction was performed on the samples using 20mL methanol and supernatant was combined into 50mL flasks and volume was made to the mark. Aliquots of the extracts were transferred to GC autosampler vials for injection on GC-FID.

A five point standard curve was generated using Δ9-THC standard (Novachem (Cerilliant) – Cat. no. T-005, Lot no. FE6212201) and CBD standard (Novachem (Cerilliant) -Cat. no. C-045, Lot no. FE1112103 at concentrations of 1.0, 0.5, 0.1, 0.05, 0.02 and 0.01 mg/mL. The concentration of cannabinoids in samples was calculated using the linear regression formula generated from the standard calibration curve.

The instrument conditions were set as below:

**Instrumentation and Setup (cannabinoid analysis):**

GC System: Agilent 6890

Column: SGE BPX5 capillary column

Column Length: 50 m

- inner diameter: 220 µm

- film thickness: 1.00 µm

Carrier Gas: Hydrogen at 1.2 mL/min

Injection Volume: 1 µL

Inlet Temperature: 280°C

Split Ratio: 20:1

Detector: Flame Ionisation Detector (FID)

- FID Temperature: 310°C

- Hydrogen Flow: 30.0 mL/min

- Air Flow: 300.0 mL/min

GC Oven Temperature Program:

1. Initial Temperature: 40°C (held for 1 min)

2. Ramp: Increase to 300°C at a rate of 10°C/min

3. Final Hold: Hold at 300°C for 10 minutes

**Instrumentation and Setup (terpenoid analysis):**

GC System: Agilent 6890

Column: SGE BPX5 capillary column

Column Length: 50 m

- inner diameter: 220 µm

- film thickness: 1.00 µm

Carrier Gas: Hydrogen at 1.2 mL/min

Injection Volume: 1 µL

Inlet Temperature: 280°C

Split Ratio: 25:1

Detector: Flame Ionisation Detector (FID)

- FID Temperature: 260°C

- Hydrogen Flow: 30.0 mL/min

- Air Flow: 300.0 mL/min

GC Oven Temperature Program:

1. Initial Temperature: 50°C (held for 1 min)

2. Ramp: Increase to 300°C at a rate of 8°C/min

3. Final Hold: Hold at 300°C for 10 minutes

6.4 Vitamin Analysis

Freeze dried powdered samples were accurately weighed (100mg) into 100mL volumetric flasks and the volume made to the mark using hexane. The samples were sonicated fro 15 mins with 10 minutes centrifugation. Aliquots were taken into autosampler vials for instrument injection.

Standard preparation – alpha-tocopherol (Sigma Aldrich Cat. No. PHR1031) was accurately weighed (25mg) into 50mL volumetric flask and the volume was made to mark with hexane. A five point standard curve was generated with concentrations of 0.5, 0.25, 0.1, 0.05 and 0.01 mg/L alpha-tocopherol.

**Instrumentation and setup**

Instrument: HPLC Agilent 1100

HPLC column: Phenomenex Silica 100A, 250 cm x 4.6mm, 3um

**Column conditions**

Wavelength: 245nm

Oven temperature: 28°C

Flow rate (mL/min): 0.75

Injection volume (uL): 10

Mobile Phase: A = Milli-Q Water, 0.05% TFA

B = Methanol, no TFA

**Table S3.** Mobile phase gradient programme

| Gradient (min) | Mobile phase A% | Mobile phase B% |
| --- | --- | --- |
| 0 | 90 | 10 |
| 10 | 90 | 10 |
| 11 | 5 | 95 |
| 16 | 5 | 95 |
| 17 | 90 | 10 |
| 22 | 90 | 10 |

B Vitamins

Freeze dried powdered samples were analysed for the presence of B Vitamins including Riboflavin (B2), Thiamine(B1), Niacin/Niacinamide (B3), Pantothenic acid ( B5) and Folic acid (B12). Samples were accurately weighed (100mg) into 100mL volumetric flasks and the volume made to the mark using 10 mg/mL ascorbic acid in Milli-Q water. The samples were sonicated for 15 mins with 10 minutes centrifugation. Aliquots were taken into auto-sampler vials for instrument injection.

Standard preparation – Riboflavin, Thiamine HCl, Niacin, Niacinamide, Folic acid and Ca-Pantothenate (Sigma Aldrich Cat. No. 47863) was accurately weighed (2.5mg) into 10mL volumetic flask and the volume was made to mark with Milli-Q Water. A five-point standard curve was generated with concentrations of 0.5, 0.25, 0.1, 0.05 and 0.01 mg/L L-ascorbic acid

**Instrumentation and setup**

Instrument: HPLC Agilent 1100

HPLC column: Phenomenex Luna C18, 250 cm x 4.6mm, 4um

**Column conditions**

Wavelength: 245nm

Oven temperature: 28°C

Flow rate (mL/min): 0.75

Injection volume (uL): 10

Mobile Phase: A = Milli-Q Water, 0.05% TFA

B = Methanol, no TFA

**6.5 Amino acid analysis**

Standard amino acid screening was performed to determine the concentration of 18 amino acid including alanine, arginine, aspartic acid, glutamic acid, glycine, histidine, hydroxyproline, isoleucine, leucine, lysine, methionine, phenylalanine, proline, serine, taurine, threonine, tyrosine, and valine using UPLC-PDA-MSMS by the [National Measurement Institute](https://www.industry.gov.au/national-measurement-institute) following ISO 13903 method guidelines. Sample were freeze dried and ground to pass through 0.5 mm sieve. An appropriate amount of sample (1 to 5g) was weighed conical flask, and the weight was recorded. To the sample, 100 mL of extraction mixture (0.1M HCl containing 2% thiodiglycol) was added and shaken for 60 minutes. The mixture was then allowed to settle, and 10 mL of supernatant was pipetted out into a 100mL beaker. Sulfosalicylic acid (5ml) was added to the beaker and stirred for five minutes, followed by removing 10mL supernatant of the resulting solution and adding it to a new beaker. The pH was adjusted to 2.20 using 1M NaOH. The resulting solution was transferred to a 50mL volumetric flask and made to the volume using 0.2M citrate buffer (pH 2.20). The extract was brought to room temperature and filtered through a 0.2 μm filter into injection vials. The analysis was then performed using HPLC equipment.
